# ROBOTIC PANCREATICODUODENECTOMY FOR THE TREATMENT OF A MIXED NEUROENDOCRINE-NON-NEUROENDOCRINE NEOPLASM (MINEN) OF THE AMPULLA OF VATER

**DOI:** 10.1590/0102-6720202400046e1840

**Published:** 2024-12-02

**Authors:** Rodrigo Cañada Trofo SURJAN, Jorge Francisco de Assis PAULINO, Henrique Perobelli SCHLEINSTEIN, Felipe Moraes Toledo PEREIRA, Estela Regina Ramos FIGUEIRA, José Celso ARDENGH

**Affiliations:** 1 Hospital Nove de Julho – São Paulo (SP), Brazil; 2 Universidade de São Paulo, Faculty of Medicine, Department of Gastroenterology, Pancreaticobiliar Surgery – São Paulo (SP), Brazil; 3 Hospital Moriah, Endoscopy Department – São Paulo (SP), Brazil.

**Keywords:** Pancreas, Robotic Surgical Procedures, Carcinoma Neuroendocrine, Pâncreas, Procedimentos Cirúrgicos Robóticos, Carcinoma Neuroendócrino

## Abstract

Mixed neuroendocrine-non-neuroendocrine tumors (MiNEN) are a rare type of tumor formed by two components, a non-neuroendocrine component that is most often an adenocarcinoma and a neuroendocrine tumor, and each of these components must represent at least 30% of the tumor. The origin of this tumor on the ampulla of Vater or periampullary region is more infrequent. Usually, the lesions are highly aggressive and quickly metastasizing, and their biological behavior is dictated by the high grade of the neuroendocrine component. This is the first report of a patient with ampullary MiNEN treated employing a robotic pancreaticoduodenectomy. Although being submitted to aggressive treatment with complete surgical resection followed by systemic therapy, the patient developed early recurrence with hepatic metastatic disease, demonstrating the hostile nature of these tumors.

## RESUMO

Os tumores mistos neuroendócrino-não-neuroendócrinos (MiNEM) são um tipo raro de tumor pancreático, formado por dois componentes: um não-neuroenócrino que geralmente é um adenocarcinoma, associado a um tumor neuroendócrino. Cada um destes componentes deve representar pelo menos 30% do tumor. A origem deste tipo de tumor na região da ampola de Vater ou região periampular é menos frequente. Geralmente, estas lesões apresentam comportamento altamente agressivo e metastatizam rapidamente, e seu comportamento biológico é ditado pelo componente neuroendócrino de alto grau. Este é o primeiro relato de um paciente com MiNEM ampular empregando a duodenopancreatectomia robótica. Apesar do paciente ter sido submetido a tratamento agressivo através de ressecção cirúrgica completa seguida de quimioterapia sistêmica, desenvolveu recidiva precoce com metástases hepáticas, o que demonstra a natureza hostil destes tumores.

## INTRODUCTION

Mixed epithelial neoplasms of the gastrointestinal tract were first described by Cordier in 1924 and are characterized by the coexistence of neuroendocrine and non-neuroendocrine histological subtypes^4^. The neuroendocrine and non-neuroendocrine components of these tumors can present different degrees of differentiation and present different types of histological arrangements, as classified by Lewin et al.^5^ in 1987: composite tumors (intimately mixed within the tumor), collision tumors (each component juxtaposed) and amphicrine tumors (with endocrine and epithelial components coexist at cellular level)^1,5^.

In 2010, the World Health Organization (WHO) considered that these tumors should contain at least 30% of each malignant component (neuroendocrine and non-neuroendocrine) as a separate entity, so called “mixed adeno-neuroendocrine carcinomas” (MANECs). Later, in 2017, the WHO renamed the pancreatic MANECs to “mixed neuroendocrine non-neuroendocrine neoplasms” (MiNENs) when the tumor origin was the pancreas, in order to better address the morphological combinations of these tumors. Finally, in the latest 2019 version, the WHO suggested the term MiNEN to all neoplasms with both neuroendocrine and non-neuroendocrine components regardless of tumor origin in the entire gastro-entero-pancreatic tract^1^.

Although MiNENs may occur in the entire digestive tract, they are rare tumors that specially affect the appendix, colon and stomach. The origin of such tumors in the ampulla of Vater and periampullary region is even more infrequent^6^. Most of the patients that present digestive tract MiNENs are males (65,6%), while only MiNENs of the appendix and biliary tract present equal proportions on gender distribution. The mean age at diagnosis is 64 years^1^.

MiNENs arising in the ampulla of Vater are not associated with specific symptoms, radiological findings and serum tumoral markers (such as CA 19-9, CEA or alpha-fetoprotein), and they are usually aggressive tumors, and timely diagnosis and treatment are crucial^6,8^. Final diagnosis is dependent on immunohistochemical examination of the tumor, specially using neuroendocrine markers such as synaptophysin, CD 56 and CgA combined with non-neuroendocrine markers as CK 20, CDX2 and cytokeratin^1^.

While there is not a consensus regarding the exact pathogenesis of these tumors and three main theories have been proposed, nowadays it is gaining more acceptance that multipotent gastrointestinal stem cells (and not cells migrated from the neural crest), such as amphicrine cells that express both exocrine and neuroendocrine components, are involved in the pathogenesis of gastrointestinal MiNENs^3^.

We describe a patient that presented with a suspected ampullary cancer. Preoperative diagnosis of an ampullary adenocarcinoma was obtained with an endoscopic ultrasound guided biopsy. The patient was submitted to a totally robotic pancreaticoduodenectomy and final pathological and immunohistochemical analysis of the surgical specimen revealed a MiNEN of the ampulla of Vater. The patient signed the informed consent to this publication.

### Surgical technique

A 71-year-old male patient with previous history of dyslipidemia, tabagism and stroke with no sequelae presented with abdominal pain and weight loss for the last three months and three days of jaundice, choluria, fecal acholia and vomiting. He had no previous abdominal surgical procedures nor familial history of digestive cancer or other risk factors relevant to the case. At hospital admission, blood tests revealed total serum bilirubin: 4,03 mg/dL (reference range: 0,2–1,2 mg/dL), alkaline phosphatase: 403 U/L (reference range: 20–40 U/L), gamma glutamyl transferase: 870 IU/L (reference range: 8–38 UI/L), alanine transaminase: 128 U/L (reference range: 4–36 U/L), CEA 1,3 ng/mL and carbohydrate antigen 19-9: 203,1 U/mL (reference range: 0–37 U/ml).

**Figure 1:**
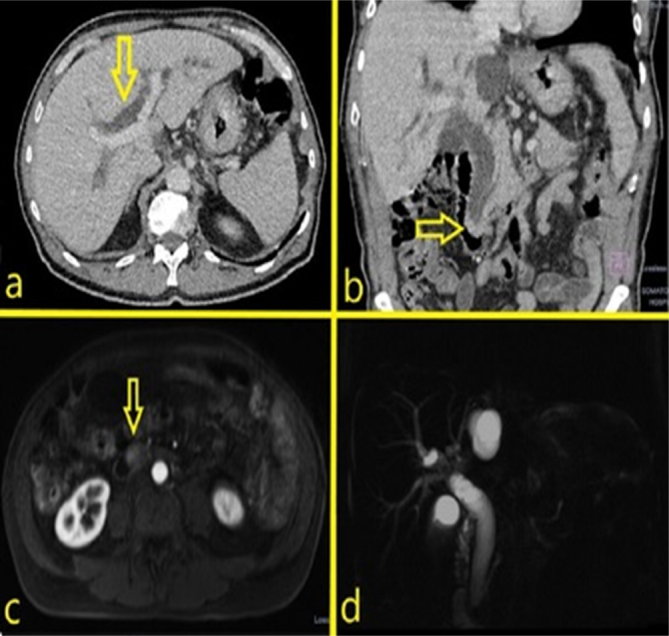
Preoperative upper abdominal computed tomography (a,b) and resonance image study (c,d). a) Contrast enhanced computed tomography, axial plane, disclosing intrahepatic bile duct dilatation (yellow arrow); b) Contrast enhanced computed tomography, coronal plane, disclosing main bile duct and pancreatic duct and nodular lesion on the ampulla of Vater (yellow arrow); c) Contrast enhanced magnetic resonance, axial plane, disclosing ampullary lesion; d) Contrast enhanced magnetic resonance, coronal plane, disclosing main bile duct, intrahepatic bile duct and main pancreatic duct dilatation.

He was initially submitted to an abdominal ultrasonography (US) that disclosed intra and extra-hepatic biliary tree dilatation. Then, an abdominal computed tomography (CT) was performed and disclosed a 1,5 cm hypervascular ampullary tumor with moderate biliary tree dilatation and mild dilatation of the main pancreatic duct (Figures 1a and 1b).

The patient was submitted to a magnetic resonance imaging with cholangiopancreatography that disclosed an ampullary lesion with minimal Wirsung dilatation and main bile duct dilatation to 2 cm (Figures 1c and 1d).

**Figure 2:**
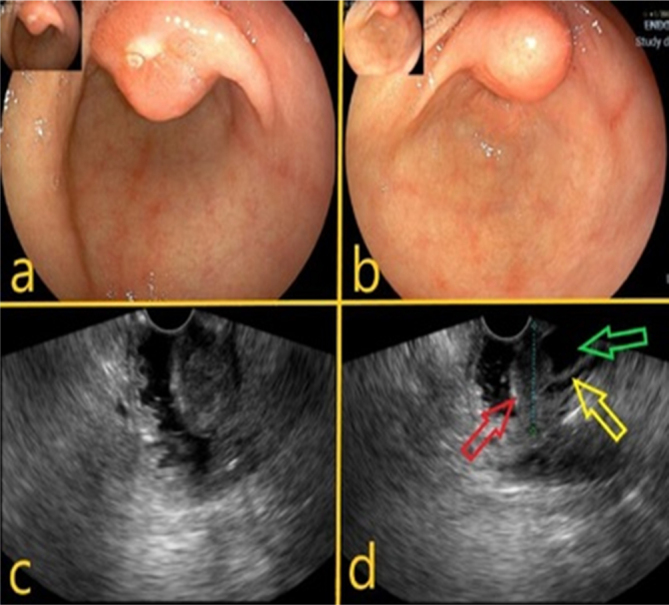
Endoscopic and endoscopic ultrasound images of major duodenal papilla. a,b) bulging and ulceration of the major duodenal papilla; c) echoendoscopic image of ampullary tumor restricted to the major duodenal papilla; d) echoendoscopic image of ampullary tumor (red arrow) and dilatation of the main bile duct (green arrow) and Wirsung duct (yellow arrow).

The option was than to perform an endoscopic ultrasound with a 20-gauge core biopsy and specimen pathological examination came out as a low-degree well-differentiated tubulovillous adenocarcinoma of the ampulla of Vater (Figure 2).

**Figure 3:**
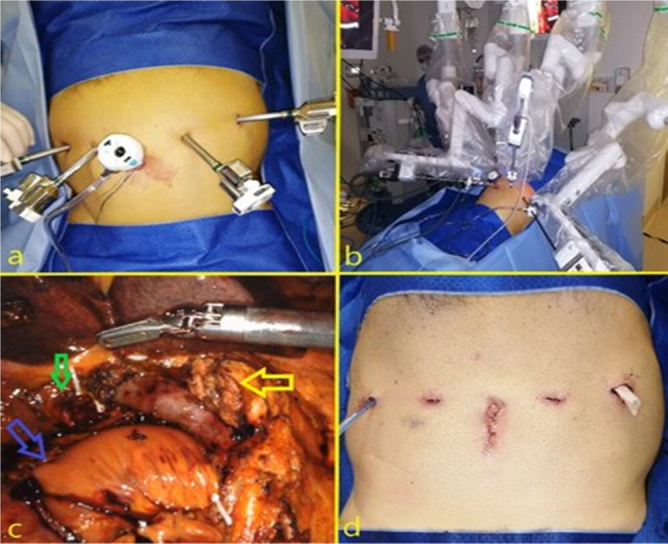
a) Trocar displacement: a 12 mm assistant umbilical port and four 8 mm robotic trocars; b) patient positioning and da Vinci Xi docking; c) intraoperative view at the end of the resection phase of the robotic pancreaticoduodenectomy (yellow arrow: pancreatic stump, green arrow: biliary stump, blue arrow: jejunal stump); d) final aspect of incisions and drains positioning.

**Figure 4:**
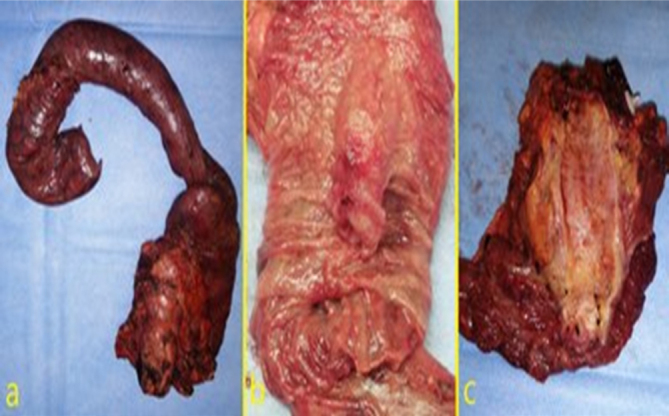
a) Surgical specimen; b) ampullary tumor; c) main bile duct and ampulla of Vater transected disclosing ampullary tumor.

As all image studies disclosed that the lesion was surgically resectable and CA 19-9 elevation was attributed to main bile duct obstruction, our option was to perform a minimally invasive totally robotic pancreaticoduodenectomy. Five trocars were used (Figure 3a). The patient was positioned in supine position with 12 degrees reverse Trendelenburg and 10 degrees left lateral tilt and the da Vinci Xi robotic platform was docked on the left side of the patient (Figure 3b). Specimen was retracted inside a plastic bag through a 2 cm enlargement of the umbilical port (Figures 3c, 3d and 4).

## RESULTS

Operative time was 8 hours, with 90 mL of estimated blood loss. Postoperative period was uneventful and the patient was discharged on the seventh day after the procedure. There was no pancreatic fistula and the pancreatic drain was removed 14 days after the surgery. Final pathology came as an ampullary MiNEN, with 60% of large cell neuroendocrine component (NEC) and 40% of epithelial intestinal type adenocarcinoma (pT1bpNo). There was no lymph node metastasis in 21 nodes retrieved. The Ki67 index was 70%.

After the procedure, the patient initiated adjuvant systemic therapy with nine cycles of FOLFOX (5-fluorouracil, leucovorin, oxaliplatin). However, the presented early recurrence with hepatic metastasis, and second line chemotherapy with 5 cycles of cisplatin + irinotecan was performed but the patient still presented metastatic disease progression. He is now (one year after the procedure) under third-line regimen with dacarbazine + 5FU (5-fluorouracil).

## DISCUSSION

Recently, new diagnostic technologies and more effective and routine use of immunohistochemical markers have allowed to shine a light on the referred grey zone of mixed tumors described by Volante et al. in 2006 and led to a better understanding of a wide spectrum of mixed tumors of the digestive tract with a broad combination of malignant components^8^. The MiNENs are rare tumors that may occur in many different sites of the digestive tract, and are characterized by a small or large cell NEC and a non-neuroendocrine carcinoma component that is always epithelial (glandular, squamous, mutinous and/or sarcomatoid). Although the pathogenesis of these tumors is unknown, several theories have been developed to explain the origins of the MiNENs. There are three important physiopathological theories that deserve to be cited:

1. Independent synchronous or metachronous development of both components from distinctive precursor cells;

2. The origin of both components by a unique pluripotent stem cell progenitor that develops biphenotypic differentiation;

3. Single monoclonal origin, that suffers stepwise trans/de differentiation of part of the epithelial component to a neuroendocrine phenotype due to molecular, genetic and micro-environmental changes^3^.

Usually, preoperative diagnosis (as in our case) of a mixed tumor by image-guide biopsy is not obtained. As demonstrated by Zhang et al., only 16% of the biopsies allow the precise preoperative diagnosis of both epithelial and NEC, as the latter usually is disposed deeper within the tumor^11^.

According to current WHO classification, MiNENs must contain at least 30% of each component at microscopic evaluation of entire surgical specimens. In up to 72,1% of the cases, there is one predominant component, usually the neuroendocrine (in 42,2% of the cases)^1^. The rationale for this arbitrarily defined threshold was originally proposed in 1987, based on the hypothesis that the prognosis is influenced by the predominant component, and a lesser than 30% represented component would not be influential in the biologic behavior of the tumor^1^. Nevertheless, this 30% threshold is frequently questioned in the literature, as even a small focus (less than 10%) of NEC can have an aggressive behavior associated with local recurrence and distant metastasis^3^.

While it is important to know the proportion of each component, usually the clinical behavior of MiNENs is determined by the NEC (high grade, most of the times) and its proliferative activity of Ki-67, being usually aggressive tumors with poor prognosis^10,11^. Therefore, adjuvant systemic therapy should usually target this component and is strongly recommended, as early local and distant recurrence has been reported in more than 50% of the patients with ampullary MiNEN^6,10^.

For resected lesions, the standard adjuvant regimen for MiNEN with NEC component has not been established. Commonly, regimens for the treatment of small cell lung cancer are used, including cisplatin, carboplatin, Camptosar (CPT-11), and etoposide are employed, but without prospective data^7^. According to a recent literature review, oxaliplatin-based combination chemotherapy is a reasonable option with less toxicity and a better security profile^10^.

A review of biliary MINEN reported that high Ki-67 index, incomplete resection, advanced tumor staging and tumoral grade were factors for poor overall survival, and that adjuvant chemoradiotherapy for those patients may contribute to better overall survival^7,9^. Thus, platinum-based combination therapy is likely to be the mainstream adjuvant chemotherapy for MINEN following radical resection for good performance patients.

We presented, to our knowledge, the first totally robotic pancreaticoduodenectomy for the treatment of an ampullary MiNEN. The robotic surgery platform is especially useful in this type of pancreatic procedures that involves delicate and precise anastomosis (such as biliodigestive and pancreaticojejunal) and prolonged operative time^2^. Despite an uneventful operative recovery and the use of adjuvant chemotherapy, the patient developed early recurrence of the tumor. This aggressive behavior is in accordance to the international literature and is dictated by the high-grade neuroendocrine component of the tumor (Ki 67: 70%).

## CONCLUSIONS

Although ampullary tumors usually have better prognosis than most biliary and pancreatic cancers, MiNENs of the ampulla of Vatter are extremely aggressive tumors with biological behavior dictated by an undifferentiated neuroendocrine component. Thus, early diagnosis and aggressive multidisciplinary treatment is crucial. We report the first totally robotic pancreaticoduodenectomy to treat an ampullary MiNEM.
